# The Reflection on the Management of Toxic Epidermal Necrolysis in the Community Setting: An Internist's Perspective

**DOI:** 10.7759/cureus.13042

**Published:** 2021-01-31

**Authors:** Qian Zhang, Ali Raza Shaikh, Thomas Yoon, Shreeja Shah, James W Mahoney

**Affiliations:** 1 Internal Medicine, Abington Hospital- Jefferson Health, Abington, USA

**Keywords:** toxic epidermal necrolysis (ten), stevens-johnson syndrome (sjs)

## Abstract

Toxic epidermal necrolysis (TEN) is a dermatological emergency that is often associated with high mortality. It is differentiated from Stevens-Johnson syndrome (SJS) based on the percentage of the total body surface area affected. There has been an established correlation with certain medications that could trigger the development of such a devastating disease. Despite numerous research studies conducted on aspects of this disease entity, TEN remains foreign to many general Internists situated in a community setting due to the extremely low disease prevalence that leads to a lack of overall experience and medical resources in dealing with this medical condition. Thus, we outlined several important management aspects of TEN/SJS that an Internist should be aware of in order to assist in prompt clinical decision making and prognosis forecasting as well as deliver effective family communication.

## Introduction

Stevens-Johnson syndrome (SJS) and toxic epidermal necrolysis (TEN) are dermatologic emergencies distinguished by severe mucocutaneous reactions with extensive necrosis and detachment of the epidermal layer of the skin, with the most common cause being medication side effects. SJS occurs when skin detachment is <10% of the total body surface area (BSA) while TEN involves >30% of BSA. TEN can also be characterized by >10% of large epithelial sheet detachment without macules. The gap of coverage involving 10-30% of skin detachment is referred to as SJS/TEN overlap [1]. These are severe conditions with high mortality as previous data estimated the mortality for each category to be 12% for SJS, 29% for SJS/TEN overlap, and 46% for TEN. Moreover, the mortality for each category increased to 24%, 43%, and 49% respectively one year after the disease onset [2]. Prior research data revealed that the incidence of SJS/TEN was 0.04% in the patients who were prescribed lamotrigine, a medication that could potentially cause severe dermatological life-threatening side effects [3]. 

## Case presentation

The patient is a 20-year-old female who presented to the emergency department (ED) with the complaint of a diffuse whole-body rash. Her past history was notable for depression as she was recently prescribed lamotrigine two weeks prior to ED presentation. She was recently discharged from another hospital two days ago where she was hospitalized for three days due to complaints of blistering of her lips, sore throat, and mild rash on the extremities. She was given the diagnosis of hand-foot-mouth disease and was treated with 1 gram of ceftriaxone. She reported a decrease in the severity of sore throat upon discharge from ED but the rash continued to progress and had spread throughout most of her body parts. The lamotrigine was subsequently discontinued by the primary care physician. She decided to present to the emergency department (ED) for further evaluation. 

**Figure 1:**
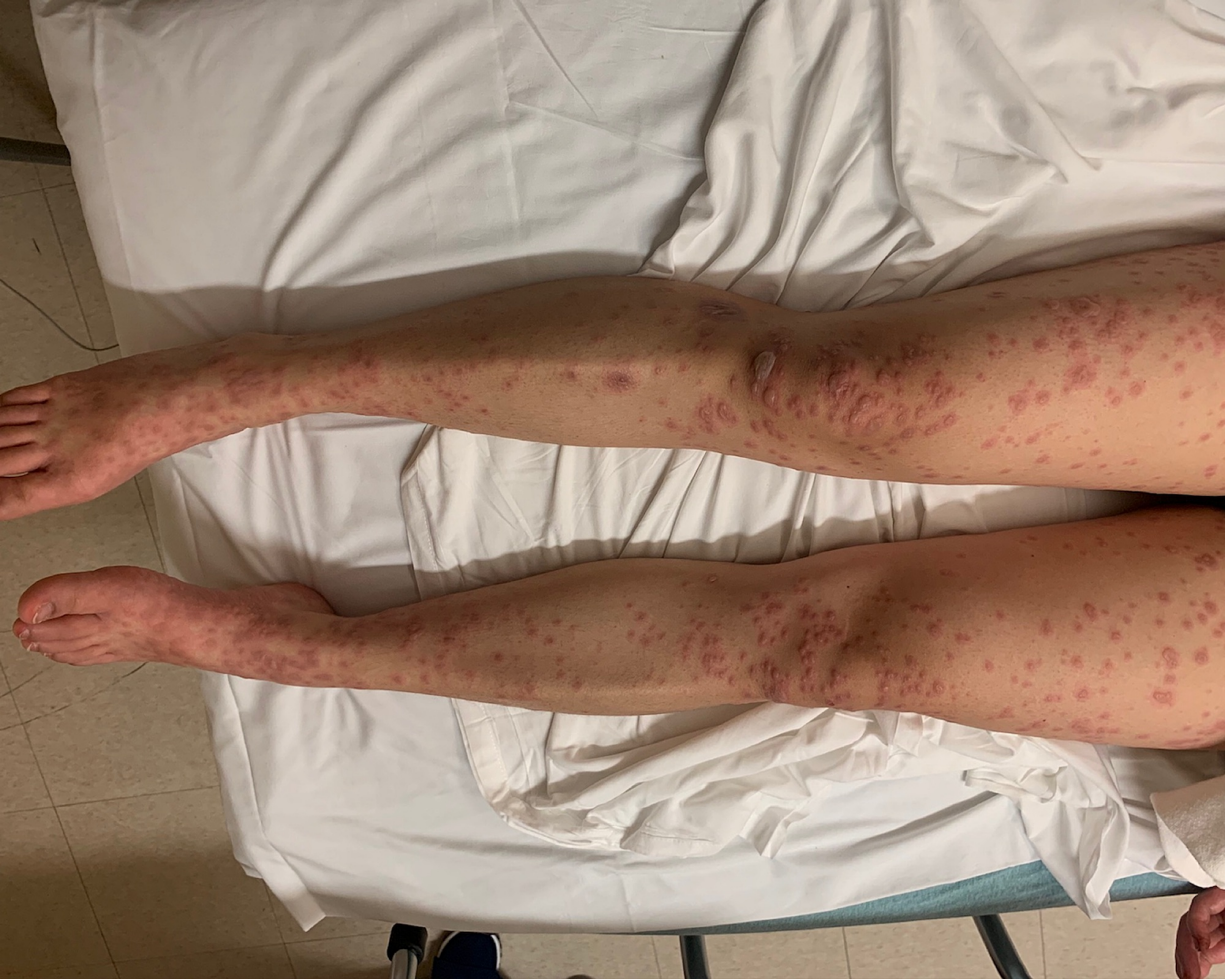
Lower Extremities

**Figure 2:**
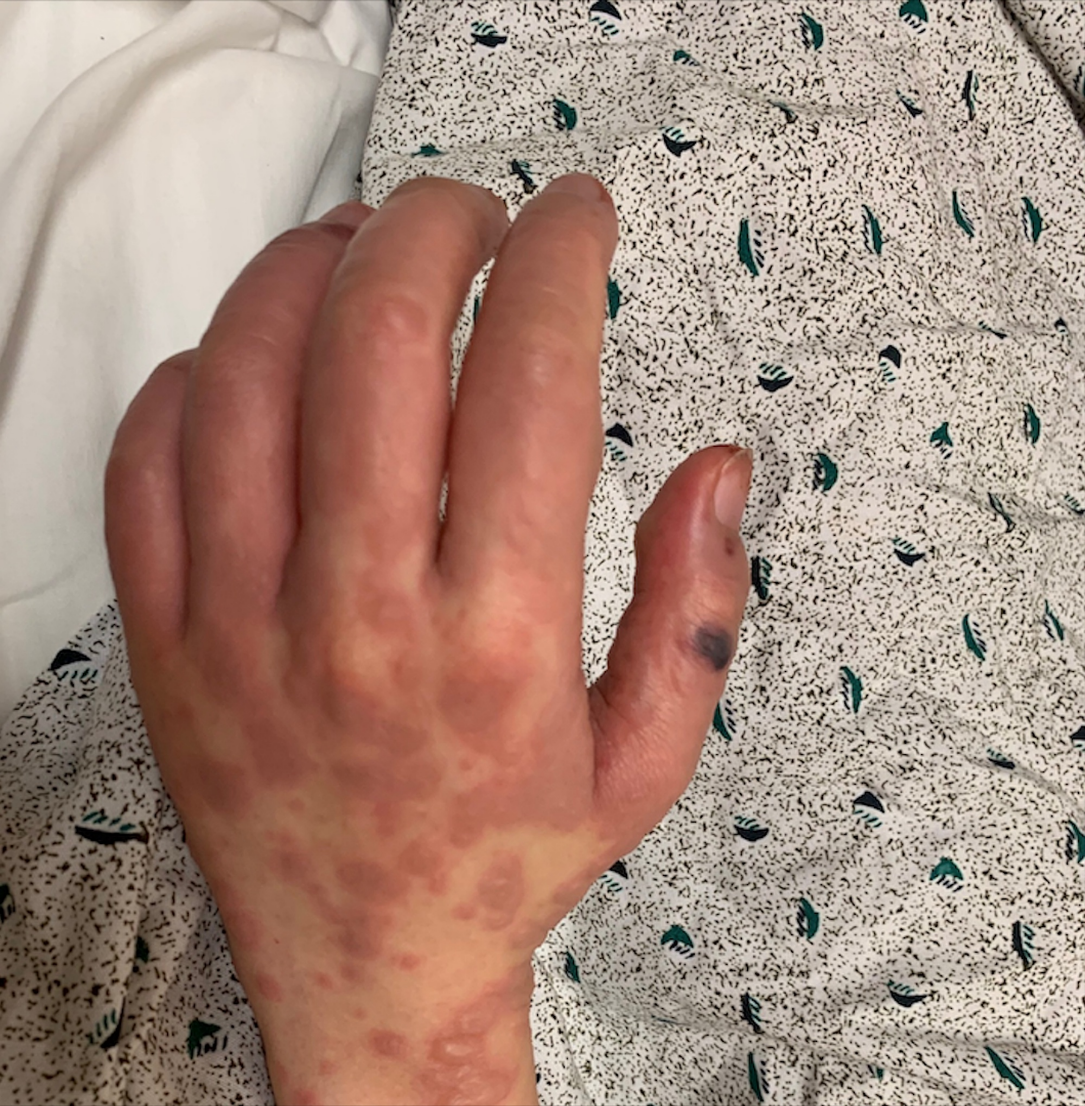
Left Hand

**Figure 3:**
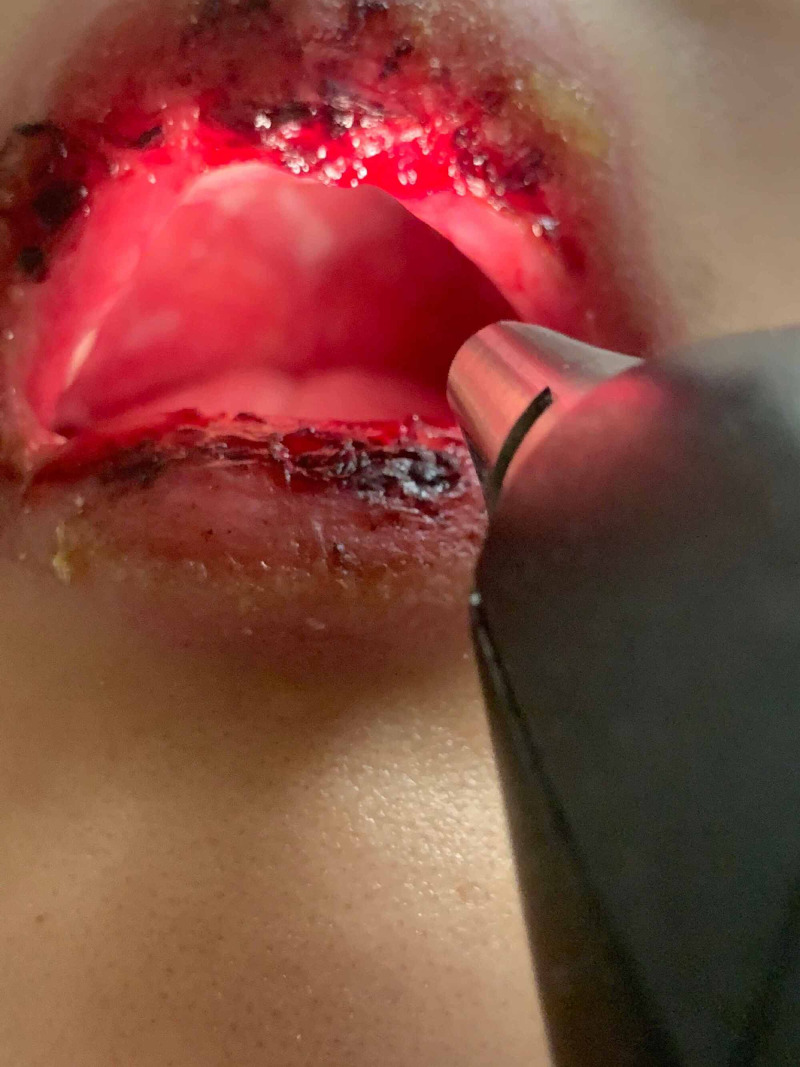
Oral Cavity

In the ED, she had a temperature of 102.4 F, blood pressure of 152/67 mmHg, heart rate of 120, and a respiratory rate of 22 per minute with an oxygen saturation of 98%. Physical examination was notable for a young female appearing uncomfortable with a diffuse maculopapular erythematous rash with scattered bullae lesions covering approximately 36% of her body surface area (Figure 1, 2). Her lips were blistered with erythematous mucus membranes as well as erythema with erosions of the eyes (Figure 3). The Nikolsky's sign was not appreciated. The rest of the physical examination was unremarkable. The laboratory data were unremarkable for signs of active infection given no leukocytosis and sterile blood cultures. Acetaminophen 1 gram was administered orally for fever and she was resuscitated with intravenous fluids. The patient was admitted to the progressive care unit for closer hemodynamics monitoring and supportive care. She was then transferred to a tertiary level burn care center within the next few hours for the continuation of care. She was diagnosed with toxic epidermal necrosis. She was monitored closely and received supportive management in the burn unit. The patient was subsequently discharged home in one week. 

## Discussion

The management of TEN could be challenging in a community setting due to the relatively low prevalence of the disease leading to the lack of experience of medical providers in dealing with this medical condition. This would generate fear and uncertainties, especially in practitioners outside of the Dermatology specialty when there is a lack of medical resources. It could further impede the prompt medical attention that the patient should receive when it comes to formulating a treatment plan, predicting prognosis, and proper communication with the patient's family members.

The ability to recognize TEN is the first step of the whole treatment process. TEN or Stevens-Johnson syndrome (SJS) patients may develop prodrome symptoms including but not limited to high-grade fever, sore throat, nonproductive cough, redness of eyes, and pink skin [4]. Mucosal lesions are an essential manifestation of TEN or SJS that could affect various body locations such as the oral cavity, ocular region, and urogenital areas [5]. Other interior organs including the trachea, bronchus, intestine, and esophagus are usually spared from the condition [6]. Once the suspicion of TEN or SJS has been raised, it is important to assess for possible triggers, most importantly certain medications that could be the culprit of the disease development. Our patient was a healthy young female with a history of depression that was treated with lamotrigine before the onset of the symptoms. The medication was promptly discontinued by the primary care physician as her condition continued to worsen. 

Prompt hospitalization is the next step in order to initiate adequate monitoring and therapy [7]. The overall treatment plan revolves around supportive management from a multidisciplinary approach. Patients with suspected TEN or SJS should be closely monitored for temperature and hemodynamics. Fluid resuscitation is important when the volume is lost due to the breakdown of the dermis. A retrospective study on patients with biopsied-proven TEN was conducted to investigate the quantity of fluids that one would require during the first 24 hours in patients with extensive areas of skin breakdown. Data from the study recommended that the quantity of fluids could be determined by multiplying the percentage of the body's skin involvement by 2 mL/kg of the body weight [8]. Furthermore, TEN patients are susceptible to various organisms that could induce sepsis or septic shock with associated mortality [9]. Studies have recommended that cultures from various sites such as blood, skin, and catheters be obtained every 48 hours [10]. Antibiotic therapy should be tailored based on the culture data. A retrospective research study reviewed 176 patients with TEN or SJS with the goal of recognizing the possible risk factors that would increase the likelihood of bacteremia. The study concluded that patients with a hemoglobin ≤10 g/dL, cardiovascular comorbidities as well as a body surface area ≥10% had a higher risk of developing subsequent bacteremia [11]. Contrary to popular beliefs, prophylactic antibiotics are not recommended despite the presence of risk factors for sepsis [12]. Wound care is also an essential component of the TEN treatment. However, it is often variable and institution dependent on the exact approach and methodology based on the resources and expertise of the hospital [13]. An urgent ophthalmologic consultation should be placed as ocular involvement could be rapidly progressive [14]. Unfortunately, there is currently a lack of adequate conclusion on the efficacy of adjunctive therapies for TEN or SJS treatment. Certain medications such as systemic corticosteroids, intravenous immune globulin (IVIG), cyclosporine, plasmapheresis, and anti-tumor necrosis factor (TNF) monoclonal antibodies are used by certain practitioners based on clinical experience with a lack of strong supporting evidence from research studies [15]. 

Community Internists should be aware of the SCORTEN scale (score of toxic epidermal necrolysis) as it could be helpful in assisting the proper disposition and prognosis of the patient. The score is composed of a total of seven variables with one point associated with each variable (≥40 years old, presence of evolving cancer or hematologic malignancies, ≥10% body area affected, heart rate ≥120/min, serum urea >10 mmol/L, serum glucose >14 mmol/L, serum bicarbonate <20 mmol/L). A higher score correlates with an increase in mortality rate and worse prognosis. It is recommended to transfer a patient with SCORTEN score ≥2 to a designated burn unit or intensive care unit for the continuation of care. In fact, a retrospective multicenter study concluded that patients who were initially treated at burn care centers had a 32% mortality rate compared to a 51% mortality rate in patients who were initially treated at a non-burn center for more than one week prior to the transfer [12]. 

## Conclusions

Patients with TEN could carry a high mortality rate if a prompt diagnosis is not made as it is crucial to position the patient as soon as possible in the inpatient setting for adequate care. Supportive management is the foundation of medical treatment for TEN in addition to the multidisciplinary approach to ensure patient safety. The utilization of the SCORTEN scale is essential for Internists to determine the proper disposition of the patient as well as to predict the likely prognosis. The awareness of timely transfer to a burn care center or the intensive care unit is especially important to practitioners situated in a community setting. 
